# A Low-cost Method to Identify Tubewells for Longitudinal Research on Arsenic in Groundwater

**Published:** 2007-09

**Authors:** Jonathan D. Sugimoto, Salahuddin Ahmad, Mahbubur Rashid, Abu Ahmed Shamim, Alain B. Labrique

**Affiliations:** 1 Department of International Health, Johns Hopkins Bloomberg School of Public Health, Center for Human Nutrition, Room W2505, 615 N. Wolfe Street, Baltimore, MD 21205, USA; 2 JiVitA Project, House 63, Road 3, Karanipara, Rangpur, Bangladesh

**Keywords:** Arsenic, Groundwater, Longitudinal studies, Plastic banding technology, Tubewell labelling methods, Tubewells, Bangladesh

## Abstract

Exposure to high concentrations of arsenic in tubewell groundwater from the shallow aquifers of Bangladesh could result in up to 300,000 arsenic-related cancer cases over the next four decades. Understanding the magnitude and temporal dynamics of this exposure, via longitudinal studies, is imperative for planning effective mitigation and management strategies. Appropriate methods are needed to identify tubewells for longitudinal sampling. A plastic band marked with a unique identification number was developed, and various methods for attaching the band to the tubewell were tested, resulting in the choice of a galvanized-iron split-rivet. Two follow-up surveys at two and 14 months post-banding assessed the durability and longevity under field conditions in the JiVitA Project area in rural, northwestern Bangladesh. After two months, ~96.0% of the original bands on 1,063 tubewells were functional, although the rivets were partially corroded. After 14 months, ~65% of a subsample of the bands were functional. With further improvements to the rivets, these bands offer an inexpensive, durable, enumeration technology for longitudinal studies on groundwater arsenic.

## INTRODUCTION

Given the described problems of arsenic contamination of groundwater from tubewells in Bangladesh (1,2), various surveys have been undertaken to quantify arsenic exposure in communities across the country (3-5). Estimates indicate that as many as 29,000,000 Bangladeshis are currently exposed to high-levels of arsenic in their drinking-water, and 200,000 to 300,000 may develop cancer as a result of this exposure (6).

Across the country there are wide, local variations in arsenic concentrations in tubewell water. Areas in the south and southeast of the country exhibit the highest levels of arsenic-content in groundwater (1). However, little is known about the degree to which the concentration of arsenic varies by season or over longer intervals of time (1,3,7). This issue needs to be investigated further since the accrued exposure and possible health consequences can vary by fluctuating arsenic contents in water. Development of tools for efficiently identifying and revisiting tubewells is a critical step to enable reliable monitoring of concentrations of arsenic and other suspected contaminants in tubewell water. Indeed, there have been calls for such a system for labelling and identifying tubewells for this purpose (8).

The International Centre for Diarrhoeal Disease Research, Bangladesh (ICDDR, B) uses field- and laboratory-based methods for testing the levels of arsenic contamination in tubewell water from their Matlab study area (9). Every test tubewell is assigned a unique nine-digit identification number. This identification number is stamped on an aluminum band, which is clipped onto the neck of the tubewell (Haq MZ. Personal communication, 2003).

When working with limited funding to plan field surveys for testing tubewells, metal-based banding technology can be expensive (ICDDR, B: ~US$ 0.13 per tubewell for 16,000 wells banded [Streatfield PK. Personal communication, 2007). A cheaper method for banding tubewells (~US$ 0.03 per tubewell for 10,000 wells banded) has been developed and tested by the JiVitA Health and Nutrition Research Project in northwest Bangladesh (Fig.) (10).

**Fig.**
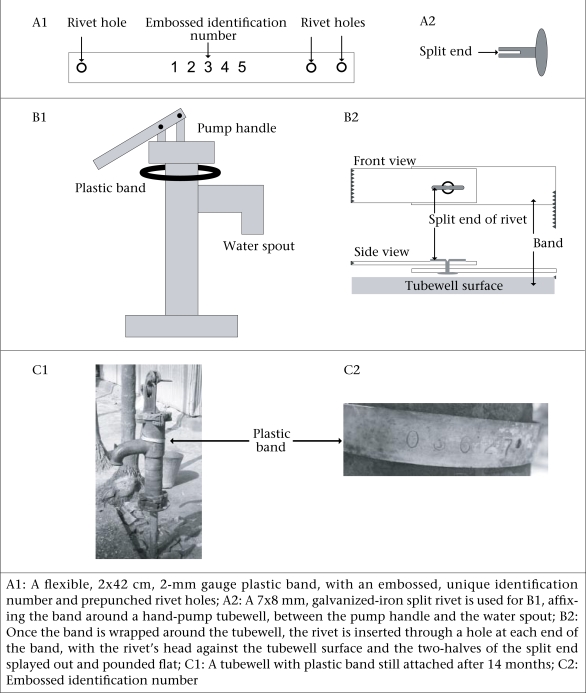
Plastic-band technology for enumerating populations of tubewells

## MATERIALS AND METHODS

The JiVitA surveillance area covers about 435 sq km, across 18 unions (subdistricts) of Gaibandha district and one union of Rangpur district. A geographic information system (GIS) covering the entire JiVitA study area was built in 2003–2004 (11). Of the ~237,000 geographic coordinates collected, ~56,400 represent tubewells.

A pilot study was conducted during May 2004 in a small subsample area of ~7.5 sq km to test various methods of tubewell banding. Bands made of sheet metal, tin, and plastic were considered but, given the number of tubewells located within the larger study area, the unit cost for a sheet metal band was too high, so they were excluded from the pilot study. Tin bands, although flexible, were not considered durable enough to withstand wear and tear under field conditions. Therefore, a flexible, twomm gauge, plastic band (2 × 42 cm) was designed with three holes for rivets—two at one end and one at the other. We produced 10,000 bands from a mold at a local plastics factory, and a five-digit tubewell identification number was embossed onto each band using a die machine (checked to ensure that no duplicate numbers were embossed).

Several potential methods for attaching the plastic bands around the neck of the tubewell (between the waterspout and the pump handle) were also considered. Riveting proved to be the most efficient method for attaching the plastic bands. Other methods, such as melting the ends of the plastic bands together with a portable blow torch or joining the ends using locally-available plastic adhesives, did not produce a strong joint. We tested several locally-available rivets, including aluminum, galvanized-iron split-rivets, and pop-rivets. Since pop-rivets cost twice as much as galvanized-iron split rivets and are more time-consuming to attach, these were excluded. At the beginning of the pilot study, aluminum rivets (corrosion-resistant) were used exclusively, but the metal of these rivets was too hard, requiring field surveyors to pound so firmly on the rivets that they consistently broke the plastic bands. As no other types of aluminum rivets were available, a switch was made to using galvanized-iron split-rivets, despite an increased risk of corrosion. To attach the band around the neck of the tubewell, the split-rivet was inserted through one hole at each end of the band—with the head of the split-rivet facing inward towards the tubewell surface. We developed a locally-produced hammer, with a wedge-shaped back end for splaying apart the two prongs of the split-end of the rivet and a square-mallet-shaped front end for flattening the splayed ends to complete the attachment process.

The bands were attached to 2,444 tubewells by a team of six GIS workers who banded tubewells, in addition to performing their normal mapping tasks of collecting GPS coordinates of the house of each subject. Tubewell banding is estimated to have taken each person one additional hour per day for 17 working days over a five-week period. The JiVitA Project conducted two cross-sectional surveys of tubewells at two and 14 months post-banding, sampled as geographically-representative, convenience samples, to estimate the percentage of bands that remained intact after these periods of time.

## RESULTS

In total, 1,063 tubewells banded with galvanized-iron split-rivets were revisited during the two-month follow-up survey (Table). Of these, 1,020 (96.0%) still had intact bands and rivets, one (0.1%) had a partially broken band, and 42 (4.0%) had broken bands or the band was missing. Of the broken bands or missing bands, five (11.9%) were missing rivets, 16 (38.1%) were the result of damage or missing tubewells, and three (7.1%) were removed by a child, and for 18 (42.9%) the cause was unknown. Thus, 96.0% (n=1,020) of the bands were still attached after two months, but all (100%) of their rivets were at least partially corroded. Tubewell banding and the first follow-up survey occurred during March-June 2004, prior to the monsoon, which is the season during which maximum corrosion of iron rivets is expected.

**Table:** Results from two follow-up surveys assessing the durability of plastic tubewell bands with galvanized-iron split rivets

	2–month survey (March 2004–June 2004)		14–month survey (March 2004–June 2005)	
Status of band	No.	%	No.	%
Tubewells visited	1,063	100.0	382	100.0
Band, intact	1,020	96.0	250	65.4
Band, partially broken	1	0.1	5	1.3
Band, broken, or missing	42	4.0	127	33.2
Missing rivet∗	5 (11.9)		81 (63.8)	
Tubewell damaged or removed∗	16 (38.1)		30 (23.6)	
Child removed band∗	3 (7.1)		1 (0.8)	
Cause unknown∗	18 (42.9)		15 (11.8)	

∗ Figures in parentheses indicate percentages of broken or missing bands

At 14 months, 382 of the 1,063 tubewells were revisited. Of these, 250 (65.4%) had intact bands and rivets, five (1.3%) had partially broken bands, and 127 (33.2%) had broken or missing bands. Of the broken or missing bands, 81 (63.8%) were the result of missing rivets, 30 (23.6%) were the result of a damaged or missing tubewell, one (0.8%) was directly removed by a child, and 15 (11.8%) were the result of an unknown cause. During the first survey, children (the most common cause of the removal of missing rivets) were reported as the direct or indirect cause of 9.5% of the missing or broken bands and 17.3% for the second survey.

## DISCUSSION

The primary drawback of plastic tubewell-banding technology is the use of galvanized-iron split-rivets. This type of rivet was found to corrode quickly under the climatic conditions of rural Bangladesh, with 100% of them being at least partially rusted (possibly accelerated by monsoon rains) after the initial two months in the field. Despite this corrosion problem, ~65% of the tubewell bands were still attached to their tubewells after more than a year. Since investigators observed that only 4% of the bands were lost after two months, the 35% loss observed after 14 months is likely to be primarily caused by environmental exposures rather than purposeful removal by users or owners. Purposeful removal of bands would have been expected shortly after the bands were first attached when they could have been viewed as novelties with value (bands are easily removed with a knife). In fact, purposeful removal was only reported for one of 382 bands at 14 months. Corrosion of rivets leading to breaks is the most plausible explanation for the loss of the plastic band.

While these findings are encouraging for short-term surveys, reflected by >95% remaining intact after two months, they suggest that, without a more durable band, the current product is not sufficiently rugged to support year-round surveillance of arsenic in tubewell water. However, since 63.8% of the broken or missing bands at 14 months appear to be due to loss of rivets, we are investigating more closely the availability of locally-produced aluminum split-rivets, which we believe would solve the corrosion problem and bring the percentage of intact bands after a year to an acceptable level (e.g. >90%).

Plastic banding is both durable and low in cost. The banding process involves minimal technology and requires little training of staff. Since these plastic bands lack commercial or utilitarian value, the likelihood of purposeful removal is low, in contrast to more versatile and valuable metal bands. With the introduction of appropriate rust-resistant rivets, this technology could find widespread use for enumerating and tracking ‘populations’ of tubewells for the longitudinal monitoring of groundwater contaminants and use in longitudinal studies of water exposure and disease in affected populations.
